# Ethanol-mediated upregulation of *APOA1* gene expression in HepG2 cells is independent of de novo lipid biosynthesis

**DOI:** 10.1186/s12944-020-01309-4

**Published:** 2020-06-20

**Authors:** Youcef Khodja, Mark E. Samuels

**Affiliations:** 1 Centre de Recherche du CHU Ste-Justine, 3175, Cote St. Catherine, Montréal, QC H3T 1C5 Canada; 2 Département de biochimie, Université de Montréal, Montreal, Canada; 3 Département de médecine, Université de Montréal, Montreal, Canada

**Keywords:** Apolipoprotein A1, APOA1, HDL, Alcohol, Ethanol, HEPG2, Liver, Cardiovascular disease

## Abstract

**Background:**

Moderate alcohol intake in human increases HDL-cholesterol, and has protective effects against cardiovascular disease (CVD). Although de novo lipid synthesis inhibitors are highly effective in lowering total and LDL-cholesterol they have only modest effects on raising HDL-C. A better understanding of the mechanism of ethanol-mediated HDL-C regulation could suggest new therapeutic approaches for CVD.

**Methods:**

Human hepatoblastoma (HepG2) and colorectal epithelial adenocarcinoma (Caco-2) cells were incubated in the presence of varying concentrations of ethanol in the culture medium, with or without addition of de novo lipid synthesis (DNLS) inhibitors (atorvastatin and/or TOFA). ApoA1 protein was measured by Western blot, and RNA of lipid pathway genes *APOA1*, *APOC3*, *APOA4*, *APOB100*, *HMGCR*, *LDLR*, and *SREBF2* by quantitative RT-PCR. Lipoproteins (VLDL, LDL, and HDL) and lipids were also monitored.

**Results:**

Ethanol stimulated ApoA1 protein (both cytoplasmic and secreted) and *APOA1* RNA levels in HepG2 cells in a dose sensitive way, with ~ 50% upregulation at 100 mM ethanol in the medium. The effect was not observed in intestinal-derived Caco-2 cells. DNLS inhibitors did not block the upregulation of ApoA1 RNA by ethanol; TOFA alone produced a modest increase in ApoA1 RNA. Ethanol had no effect on ABCA1 protein levels. Addition of ethanol to the cell medium led to modest increases in de novo synthesis of total cholesterol, cholesteryl esters and triglycerides, and as expected these increases were blocked when the lipid synthesis inhibitors were added. Ethanol stimulated a small increase in HDL and VLDL but not LDL synthesis. Ethanol in the cell medium also induced modest but measurable increases in the RNA of *APOC3*, *APOA4*, *APOB*, *LDLR*, and *HMGCR* genes. Unlike *APOA1*, induction of RNA from *APOC3* and *APOA4* was also observed in Caco-2 cells as well as HepG2 cells.

**Conclusion:**

This study has verified the previously reported upregulation of *APOA1* by exposure of HepG2, but not Caco-2 cells, to ethanol in the culture medium. It is shown for the first time that the effect is dependent on RNA polymerase II-mediated transcription, but not on de novo biosynthesis of cholesterol or fatty acids, and therefore is not a generalized metabolic response to ethanol exposure. Some other lipid pathway genes are also modulated by ethanol exposure of cells. The results reported here suggest that the proximal signaling molecule leading to increased *APOA1* gene expression in response to ethanol exposure may be free acetate or acetyl-CoA.

**Take home:**

Upregulation of ApoA1 gene expression in hepatoma cells in culture, upon exposure to moderate ethanol concentrations in the medium, occurs at the level of RNA and is not dependent on new cholesterol or fatty acid synthesis. The primary signaling molecule may be free acetate or acetyl-CoA. These results are important for understanding the mechanism by which moderate alcohol consumption leads to upregulation of serum HDL-cholesterol in humans, and suggests new approaches to targeting HDL as a risk factor for cardiovascular disease.

## Introduction

Cardiovascular disease (CVD) is a leading cause of morbidity and mortality around the world [1, 2]. Higher levels of high density lipoprotein cholesterol (HDL-C) and its major protein constituent, apolipoprotein A-1 (ApoA1) have been associated with lowered risk of cardiovascular diseases in many studies performed in diverse ethnic populations [3–8]. In contrast, low levels of circulating HDL-C and ApoA1 are positive risk factors for atherogenesis and for overall mortality. Consumption of moderate amounts of alcohol is also well documented to be cardioprotective. The epidemiology of alcohol, specifically ethanol, typically demonstrates a U-shaped or J-shaped curve, in which moderate ethanol consumption reduces mortality and CVD in comparison both to abstinence and heavier consumption [9–14]. In general these studies show similar effects independent of the type of alcohol consumed (beer, wine, distilled liquors), although additional protective effects of anti-oxidants in red wines have also been studied [14, 15].

The mechanisms by which ethanol exerts a cardioprotective effect remain poorly understood. At least some of the protective effect of ethanol appears to be through changes in circulating lipids and haemostatic factors [15–20]. Specifically, approximately 50% of the protective action of alcohol consumption is reportedly mediated by an increase of HDL-C [21, 22]. The recommended daily rate of alcohol consumption leads to a mean increase of plasma HDL-C of 0.07-0.1 mM. This amount seems small, but is equivalent to 5–10% of the mean HDL-C in the general population, thus represents a significantly improved lipoprotein profile [9, 11].

Despite the strong interest in HDL physiology, and the importance of understanding the effects of ethanol consumption, the specific biochemical mechanism by which ethanol raises circulating HDL-C is not understood, and indeed has not been extensively studied. Orally consumed ethanol is rapidly metabolized in the liver, yielding primarily acetate and acetyl-CoA [23, 24]. Isotope studies have shown that a significant proportion of ingested ethanol is ultimately released as CO_2_, presumably through the tricarboxylic acid cycle and oxidative respiration [25]. There is clearly a need for simpler model systems, either in cell culture or animals, to tease apart the various strands of ethanol physiology with regard to HDL and cardiovascular disease.

There is a small literature addressing some of these questions. Amarasuriya et al demonstrated that addition of ethanol to the culture medium of human hepatocyte-like (HepG2) cells led to an increase in ApoA1 protein synthesis [26]. A handful of subsequent studies by several groups confirmed this finding and extended it to other lipoproteins synthesized by these cells [27–29]. Somewhat surprisingly the molecular mechanism of this stimulated level of expression, and indeed the identity of the proximal signaling molecule, has not been determined. Although ethanol is typically metabolized via alcohol dehydrogenase, this activity is not expressed in HepG2 cells, leaving the microsomal ethanol oxidizing system (MEOS) as the major pathway of ethanol catabolism to acetaldehyde [28, 30, 31]. The induction of ApoA1 by ethanol was blocked in HepG2 cells by metyrapone, an inhibitor of the MEOS, suggesting that the immediate signal is not ethanol itself [28]. To the best of our knowledge these findings have lain dormant for the past two decades.

The goal of this study was to verify the earlier findings in cultured hepatoblastoma cells, and to address the mechanism by which ethanol stimulates *APOA1* gene expression. It is shown that currently available HepG2 cells demonstrate the observed effect on *APOA1*, both at the protein and RNA level, and that the RNA upregulation requires RNA polymerase II activity. The results are consistent with an effect on transcription of the *APOA1* gene. Further, it is shown for the first time that the upregulation is independent of de novo synthesis of cholesterol or fatty acids. These results suggest that the proximal signaling molecule may be free acetate or acetyl-CoA.

## Materials and methods

TOFA (5-(tetradecycloxy)-2-furoic acid) was from Abcam (Toronto; ON, Canada). Sodium acetate was from Sigma-Aldrich (Oakville;ON, Canada). (3S, 5S)-atorvastatin sodium salt was from My BioSource (San Diego; CA, USA). Oleic acid-albumen, BSA and α-amanitin were from Sigma (Oakville;ON, Canada). Dulbecco’s Modified Eagle Medium (DMEM), Minimum Essential Medium (MEM), fetal bovine serum (FBS), L-glutamine (200 mM), penicillin/streptomycin (10,000 Units/mL and 10,000 μg/mL, respectively), and 0.5% trypsin-EDTA-10X were from Gibco Thermofisher Scientific (Ottawa; ON, Canada). Hu-LPDS was from Millipore (Temecula-California). Anti-ApoA-I and anti-mouse IgG HRP- linked antibodies were from Cell Signaling technology (CST). Anti-beta actin antibodies were from Novus Biologicals (Centennial; CO, USA). Protease inhibitor cocktail and PMSF were from Roche, ethanol 100% was from Greenfield, Inc. (Ontario, Canada), trypan blue was from Thermofisher Scientific (Ottawa; ON, Canada).

### Cell culture

Human hepatocellular carcinoma cells (HepG2) were freshly obtained from the ATCC (Manassas, VA). Cells were cultured in 10-cm^2^ culture dishes containing 1 mL of culture medium per cm^2^. Unless stated otherwise the standard medium was Dulbecco’s Modified Eagle Medium (DMEM) containing 10% fetal bovine serum (FBS), penicillin and streptomycin (10,000 units /mL and 10,000 μg /mL respectively). One week before the start of experiments, cells were split at a ratio of 1:6 and seeded into 6-well plates at a density of about 10^5^ cells/well in 2 mL standard medium. The medium was replaced after 3 days.

Human colorectal adenocarcinoma cells, (CaCo-2) were kindly provided by Dr. Ali Ahmed. Cells were cultured in Eagle’s minimal essential medium (EMEM) containing 10% FBS, L-glutamine, and penicillin/streptomycin (10,000 units/mL and 10,000 μg/mL respectively). HepG2 and Caco-2 cells were maintained at 37 °C in a saturating humidity atmosphere containing 95% air and 5% CO_2_. At the start of the incubations the cells were grown to confluence.

Experiments with test compounds were carried out in DMEM plus 10% FBS or 3 mg/mL of human lipoprotein deficient serum (LPDS) from Millipore (Etobicoke, ON, Canada). In some experiments the medium was DMEM (Gibco), with 1000 mg glucose/L supplemented with penicillin/streptomycin, non-essential amino acids and bovine acid oleic-albumin (BAOA). Single compounds were added from concentrated stock solutions in water or DMSO as appropriate.

### HepG2 and Caco-2 incubation with ethanol or sodium acetate

HepG2 or Caco-2 cells were cultured in 6-well plates and incubated for varying times in the presence of final media concentrations of 0, 10, 25, 50 or 100 mM ethanol. The culture medium in each plate was changed every 24 h in induction experiments of longer duration. For the experiments in which ApoA1 RNA was measured, HepG2 and Caco-2 cells were incubated with varying concentration of ethanol (0–500 mM) for 24 h. For the experiments in which ABCA1 protein was measured, HepG2 cells were incubated with ethanol (50 or 100 mM) for 24 h. For the acetate experiments, HepG2 cells were incubated with medium containing sodium acetate (0, 5 or 10 mM) for 24 h. Cell death was assessed using trypan blue exclusion and was minimal in all experiments except in the presence of 500 mM ethanol or 10 mM sodium acetate.

### HepG2 incubation with ethanol plus α-amanitin, atorvastatin or TOFA

HepG2 cells of approximately 70–80% confluence were grown for 24 h in standard medium media prior to experiments. At the initiation of incubations, cells were washed twice with phosphate buffered saline (PBS). Then the experimental medium plus DMEM with 10% FBS, or 3 mg/mL of LPDS, was added, containing variously either α-amanitin (20 uM), atorvastatin (20 uM) or TOFA (20 uM) alone, or in combination with ethanol, at varying concentrations as indicated in the relevant figure legends. In some experiments the experimental medium was DMEM (Gibco), also containing 1000 mg glucose/L) supplemented with penicillin/streptomycin, non-essential amino acid and BSA (Sigma), and different concentrations of TOFA and ethanol, or TOFA together with ethanol were added. Atorvastatin sodium salt and TOFA stock solutions were made with DMSO as solvent. An equivalent volume of DMSO was always added to control cells. Unless stated otherwise, cells were incubated at 37 °C for 24 h.

### ApoA1 and ABCA1 protein measurement

ApoA1 protein levels were measured by Western blot. Cells were lysed by RIPA buffer (25 mM Tris-HCl pH 7.5, 5 mM NaCl, 0.5 mM EDTA, 0.1% SDS, 1% Triton X-100 (BioShop; Burlington, ON; Canada), and a protease/PMSF inhibitor cocktail. Lysates were centrifuged at 14,000 × rpm for 10 min at 4 °C in a fixed angle rotor FA-45-24-11, and the supernatant was collected. For measuring ApoA1 protein in conditioned medium, 1 mL of medium was precipitated with trichloroacetic acid (TCA) according to the manufacturer’s protocol (Sigma). Protein concentration was measured with a Quick Start™ BSA kit (Bio-Rad; Mississauga, ON, Canada). Cell lysates and protein precipitates with TCA were dissolved for electrophoresis in Laemmli sample buffer and incubated for 5 min at 95 °C, pH adjusted as needed with Tris-HCl. Samples (30 μg of total protein per well) were fractionated by electrophoresis in a 10% (wt/vol) SDS-polyacrylamide gel and transferred to a nitrocellulose membrane 0.45 μm (Bio-Rad). The membrane was blocked for 1 h with 5% (wt/vol) skim milk powder in TBS-Tween (T-TBS, Tween from BioShop). Membranes were incubated overnight at 4 °C with monoclonal primary antibody (anti-ApoA1 at 1:1000 dilution, cat. SAB1410670; anti-ABCA1 at 1:2000 dilution, cat NB100–2068; or anti-B-Actin at 1:5000 dilution; cat NB600–501 (Novus Biologicals), in 2.5% (vol/vol) blocking solution in T-TBS. After three washes with T-TBS, detection was performed by using goat anti-rabbit IgG H&L (cat. no ab 6721; Abcam), goat anti-mouse IgG (cat HAF007; Novus Biologicals), or alpha-mouse (cat. No ab 7075; Abcam) at dilutions of 1:3000, 1:5000, or 1:5000 respectively. Quantification was by the enhanced chemiluminescence reagent system (Pierce ECL, Thermofisher Scientific), using CCD camera-based imaging (GE Healthcare; Bio Science). For measurements of cytoplasmic ApoA1 protein, results were normalized to beta-actin in each lane and analyzed by ImageJ software. Due to the very large difference in concentration of ApoA1 or ABCA1 and actin, the same blots were probed and photographed separately with antibodies against the two proteins. For measurements of secreted ApoA1 protein, equivalent volumes of cell culture medium were TCA-precipitated as there is no consensus on an internal standard for secreted protein samples.

### Total RNA extraction and real time (RT)-PCR

At the indicated time after incubation with medium plus ethanol or other reagents, total cell RNA was extracted using TRIzol (Invitrogen,Carlsbad, CA), according to the manufacturer’s instructions. RNA was electrophoresed on a 2100 Bioanalyzer using a Nano RNA chip to verify its integrity. Total RNA was treated with DNase and reverse transcribed using the Maxima First Strand cDNA synthesis kit with dsDNase (Thermo Scientific). Primer Assay Design Centre (https://lifescience.roche.com/en_ca/brands/universal-probe-library.html#assay-design-centre) was applied to design the primers and probes of human *APOA1, APOC3, APOA4, APOB, HMGCR, LDLR, SREBF2, TBP* and *GAPDH* (the sequences of primers and probes are shown in Table 1. RNA was determined by quantitative RT-PCR (qPCR) using assays designed with the Universal Probe Library from Roche. For each qPCR assay, a standard curve was performed to validate the dynamic range of the assay. qPCR reactions were performed using Perfecta QPCR FastMIX II (Quanta), 2 μM of each primer and 1 μM of the corresponding UPL probe. The Viia7 qPCR instrument (Life Technologies) was used to detect the amplification level and was programmed with an initial step of 20 s at 95 °C, followed by 40 cycles of: 1 s at 95 °C and 20 s at 60 °C. Relative expression (RQ = 2-ΔΔCT) was calculated using the Expression Suite software (Life Technologies), and normalization was done using both GAPDH and TBP as internal control housekeeping genes. Formally, this analysis measures total not cytoplasmic mRNA for the assayed genes.

**Table 1:** Sequences of primers used to amplify the indicated genes for qRT-PCR measurement of RNA levels in treated cells

Oligo ID	Gene	UPL Probe	Oligo FWD	Oligo REV	RefSeq
**IR4651**	**APOA1**	**39**	ccttgggaaaacagctaaacc	ccagaactcctgggtcaca	NM_000039.1
**IR4652**	**APOC3**	**3**	gccaaggatgcactgagc	gaactgaagccatcggtcac	NM_000040.1
**IR4653.2**	**ApoA4**	**41**	ccagggctgaggtcagtg	tgtcctggaagagggcatt	NM_000482.3
**IR5265**	**ApoB100**	**90**	acagctgattgaggtgtcca	agccactggaggatgtgagt	NM_000384.2
**IR5263**	**LDLR**	**11**	tcggctacgagtgcctgt	gatcctgacactcatcgatatcttc	NM_001195798.1, NM_001195799.1, NM_001195800.1, NM_001195803.1, NM_000527.4
**IR4714**	**HMGCR**	**85**	gacgcaacctttatatccgttt	ttgaaagtgctttctctgtaccc	NM_000859.2 NM_001130996.1
**IR5264**	**SREBF2**	**82**	caccggaaacaggcagat	ccaggcaggtttgtaggttg	NM_004599.2
**IR3024**	**GAPDH**	**60**	agccacatcgctcagacac	gcccaatacgaccaaatcc	NM_002046.3
**IR3658**	**TBP**	**87**	gaacatcatggatcagaacaaca	atagggattccgggagtcat	NM_003194.3

### Measurement of lipid synthesis and secretion

Lipid synthesis and secretion were assayed as previously described [32, 33]. Briefly, radiolabeled [^14^C]-oleic acid (specific activity 59.0 mCi/mmol; PerkinElmer, Boston, US) was added to unlabeled oleic acid-BSA (Sigma). The final oleic acid concentration was 0.7 mM (0.45 μCi)/well. Cells were first washed with PBS, and the [14C]-oleic acid-containing medium was added to the upper compartment. At the end of a 24-h incubation period, cells were washed, and then scraped with a rubber policeman in RIPA buffer containing anti-proteases (pepstatin, leupeptin, PMSF all at a final concentration of 1 mM). An aliquot was taken for lipid extraction by standard methods in the presence of unlabeled carrier (phospholipids, triglyceride and cholesteryl esters).

The various lipid classes synthesized from [^14^C]-oleic acid were separated by thin-layer chromatography (TLC) using the solvent mixture of hexane, ether, and acetic acid (80:20:3, vol:vol:vol), as previously described [32, 33]. The area corresponding to each lipid was scraped off the TLC plates, and the silica powder was placed in a scintillation vial with Ecolite (+) liquid scintillation cocktail (MP Biomedicals, CA). Radioactivity was then measured by scintillation counting (Hidex 300 SL). Cell protein was quantified as described above, and results were expressed as disintegrations per min (dpm) per milligram of cell protein. Lipid secreted in the basolateral compartment was analyzed and quantified, as described above, after centrifugation (2000 rpm for 30 min at 4 °C) to remove cell debris.

### Lipid carrier

Blood was drawn 3 h after the oral intake of a fatty meal by a human volunteer, and postprandial plasma was prepared to serve as a carrier for the lipoproteins synthesized by HepG2 cells. The TG-enriched plasma was incubated at 56 °C for 1 h to inactivate enzymatic activity in the presence of anti-proteases.

### Isolation of lipoproteins

For the determination of secreted lipoproteins, HepG2 cells were incubated with the lipid substrate as described above. The medium supplemented with anti-proteases (as described above) was first mixed with a plasma lipid carrier (4:1, vol:vol) to efficiently isolate de novo lipoproteins synthesized. The lipoproteins were isolated by sequential ultracentrifugation using a TL-100 ultracentrifuge (Beckman). Briefly, chylomicrons were isolated after ultracentrifugation (25,000 rpm for 40 min). Very low-density lipoprotein (1.006 g/mL) and low-density lipoprotein (LDL, 1.063 g/mL) were separated at 90,000 rpm for 2 h and 47 min in a tabletop ultracentrifuge 100.4 rotor at 4 °C. The high-density lipoprotein fraction was obtained by adjusting the LDL infranatant to a density of 1.21 g/mL by adding Kbr followed by centrifugation for 7 h and 15 min at 90,000 rpm. Each lipoprotein fraction was exhaustively dialyzed against 0.15 M NaCl and 0.001 M EDTA, pH 7.0, at 4 °C for 24 h.

### Statistical analyses and software

Results are presented as means ± standard deviation. All experiments were repeated at least three times or five times with independent biological replicates. For assessing cell vitality individual biological experiments were performed as duplicates, respectively. Group analyzes were performed using one-way two-way analysis of variance (ANOVA) by GraphPad Prism 6.04 (GraphPad, La Jolla, CA, USA). P-alues < 0.05 were considered statistically significant.

## Results

### Ethanol induces *APOA1* expression in HepG2 cells

**Fig. 1:**
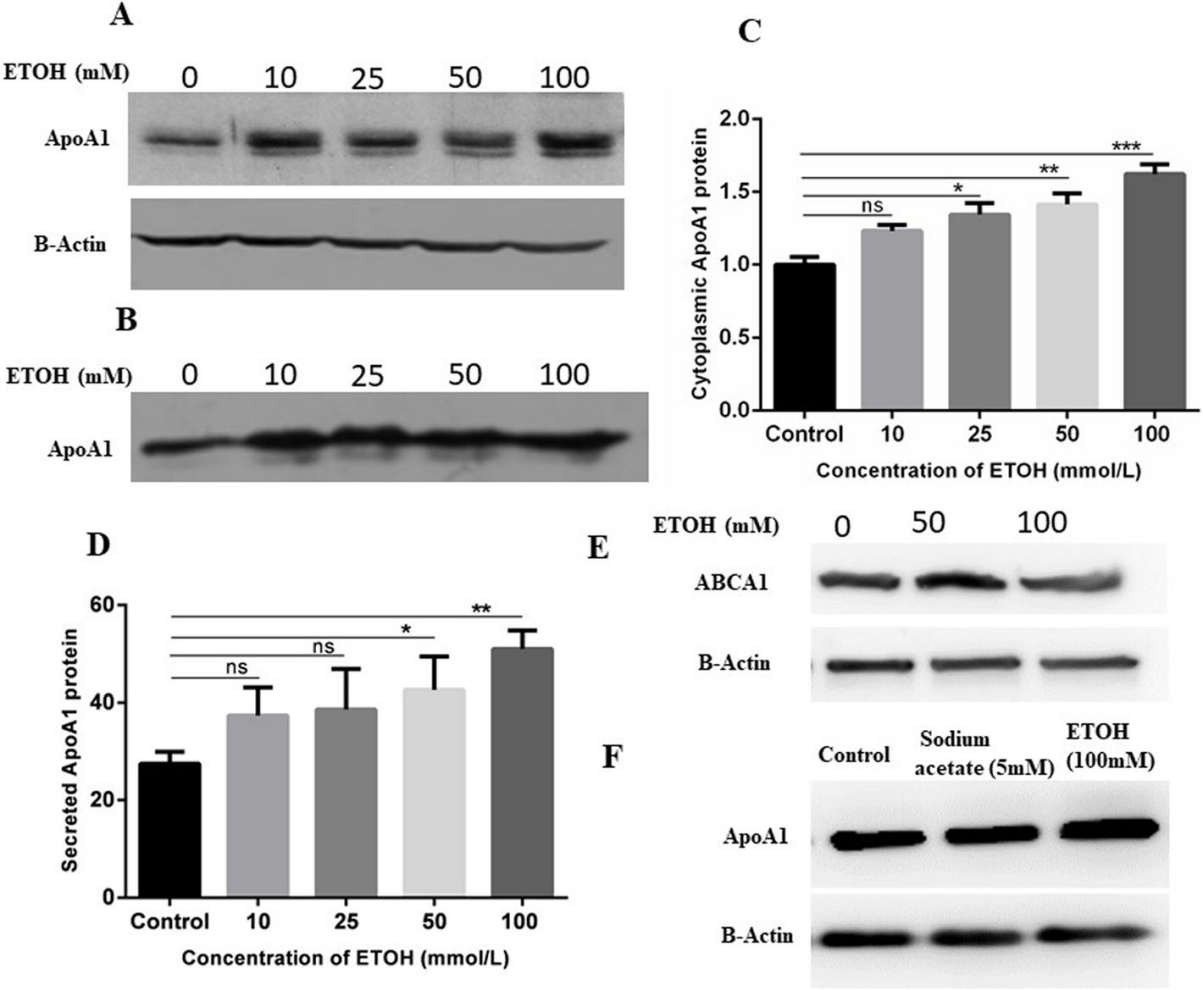
ApoA1 and ABCA1 protein expression in cells treated with ethanol or acetate. HepG2 cells were incubated in standard medium with addition of the indicated concentration of ethanol (0, 10, 25, 50, 100 mM). Cell lysates (panel **a**) or TCA-precipitated culture medium (panel **b**) was analyzed by Western blot to detect ApoA1 protein. Actin protein was used to control for loading of cytoplasmic extracts, equivalent volumes were precipitated to control for loading of protein precipitated from the culture medium. In all cases plates contained similar densities of cells. **c** Quantified cytoplasmic ApoA1 protein relative to β-actin averaged from three independent experiments. **d** Quantified precipitated ApoA1 protein from culture mediums, averaged from three independent experiments. **e** HepG2 cells were treated with the indicated concentrations of ethanol for 24 h, and cell extract were ABCA1 protein was assayed by Western blot. **f** HepG2 cells were treated with the indicated concentrations of ethanol or sodium acetate for 24 h, and cell extract ApoA1 protein was assayed by Western blot. Data are expressed as means ± SD of three experiments for ApoA1 protein and RNA expression (1 way ANOVA with multiple comparisons; *P < 0.05, **P < 0.01, ***P < 0.001 vs. control

As an important preliminary first step, it was confirmed that currently available hepatocyte-like cells would respond to ethanol as previously published, as it is well known that the same cell lines may vary considerably between laboratories, and that immortalized cell lines may evolve over many generations of passage. Fresh HepG2 cells were obtained from the ATCC catalog. As shown in Fig. 1, total ApoA1 protein levels increased in a dose-dependent manner with increasing ethanol concentration in the medium, both in cell cytoplasm (panels A, C), and as secreted into the culture medium (panels B, D). Thus currently available HepG2 cells demonstrate the previously reported behavior in response to ethanol in the culture medium.

Since ethanol is rapidly metabolized, via acetate, to acetyl-CoA, the effect of adding acetate to the culture medium was also explored. 10 mM sodium acetate in the medium led to cell death, therefore 5 mM acetate was employed, a concentration which was previously shown to upregulate lipogenic gene expression in hypoxic cells [34, 35]. However, no effect on ApoA1 protein levels was observed in the treated cells (Fig. 1f). Note that all cells were grown under standard normoxic conditions.

**Fig. 2:**
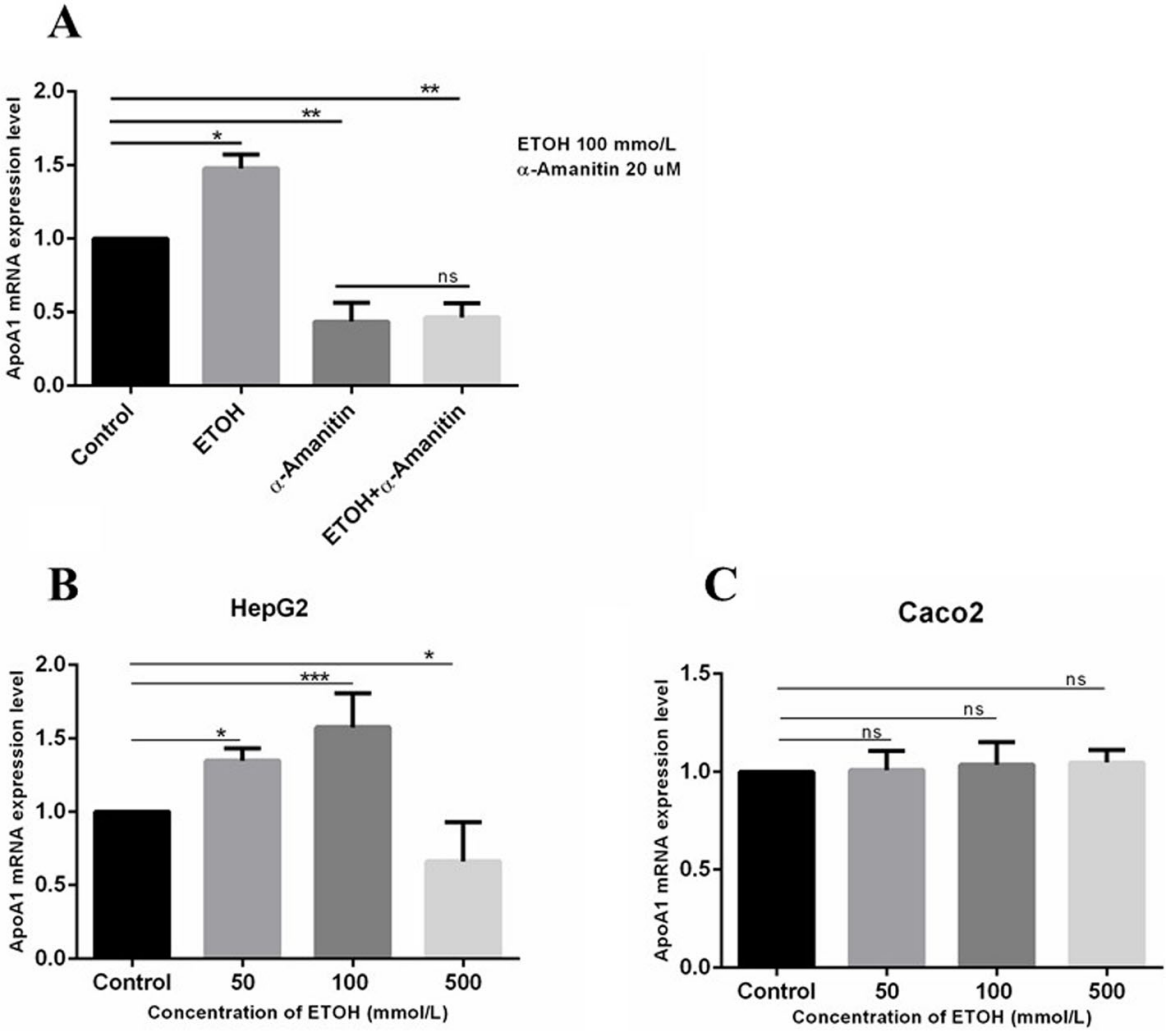
*APOA1* RNA is upregulated by ethanol and requires RNA polymerase II activity. Relative RNA expression levels were determined by RT-qPCR as described in Methods. HepG2 were cultured to ~ 80% confluence on six well plates before treating with ethanol. **a** HepG2 cells were exposed to 100 mM ethanol and/or α-amanitin as per Materials and Methods. **b** and **c** Relative RNA expression levels were determined by RT-PCR as described in Methods. Dose response curve of *APOA1* RNA at the indicated final concentrations of ethanol in the medium. The error bars represent the SD from the mean of five assays of an individual experiment. *Significant difference, ethanol treatment versus untreated control (1 way ANOVA with multiple comparisons, *n* = 5; *P < 0.05, **P < 0.01, ***P < 0.001 vs. control)

The previous findings on *APOA1* RNA expression were significantly extended using quantitative RT-PCR. As seen in Fig. 2a, b, ethanol increased the level of *APOA1* RNA in the cells when compared with the control cells not receiving ethanol in a dose sensitive response up to 100 mm, above which RNA levels decreased probably due to ethanol toxicity at the higher concentration. This finding is very robust, having been repeated in numerous experiments each with multiple plate replicates. A time course showed that *APOA1* RNA increase was first observable 12 h after addition of ethanol to the culture medium (data not shown). The maximum increase in *APOA1* RNA was typically 50% above the untreated cell baseline.

In order to see if the induction of *APOA1* expression by ethanol was specific to liver-derived cells, intestinal-derived Caco-2 cells were treated with different concentrations of ethanol (from 50 mM to 500 mM). No increase in *APOA1* expression was observed in these cells (Fig. 2c).

### Upregulation of *APOA1* RNA requires RNA polymerase II activity

To examine the cellular level of RNA expression modulated by ethanol, HepG2 cells were treated with ethanol alone (100 mM) or with ethanol plus α-amanitin. α-Amanitin is a cyclic peptide isolated from *A. phalloides* mushrooms, that strongly inhibits RNA polymerase II at low concentrations. It can be added to cell culture medium, and is taken up by cells where it rapidly inhibits de novo transcription by Pol II. Effects on gene expression which depend on new transcription are therefore blocked by addition of α-amanitin. Quantitative real time PCR was performed on the total extracted RNA. The addition of α-amanitin prevented the induction of *APOA1* expression (Fig. 2a). Amanitin reduced the total level of *APOA1* RNA in the presence or absence of ethanol; this is expected presuming the drug inhibits both basal and induced expression.

### *APOA1* upregulation by ethanol is not dependent on de novo lipid synthesis

As mentioned in the Introduction, the fate of all the carbons in ingested ethanol is uncertain though the majority is ultimately converted to CO_2_ via respiration. It has been reported that a small fraction of ethanol may be used for de novo lipid synthesis (DNLS) in hepatocytes in vivo and in culture [36]. To address whether these processes are relevant to the observed upregulation of *APOA1* RNA, two inhibitors of lipid synthesis were tested for effects (if any) on ethanol induction of *APOA1* expression. Specifically, atorvastatin and TOFA were used to block DNLS of cholesterol or fatty acids respectively.

**Fig. 3:**
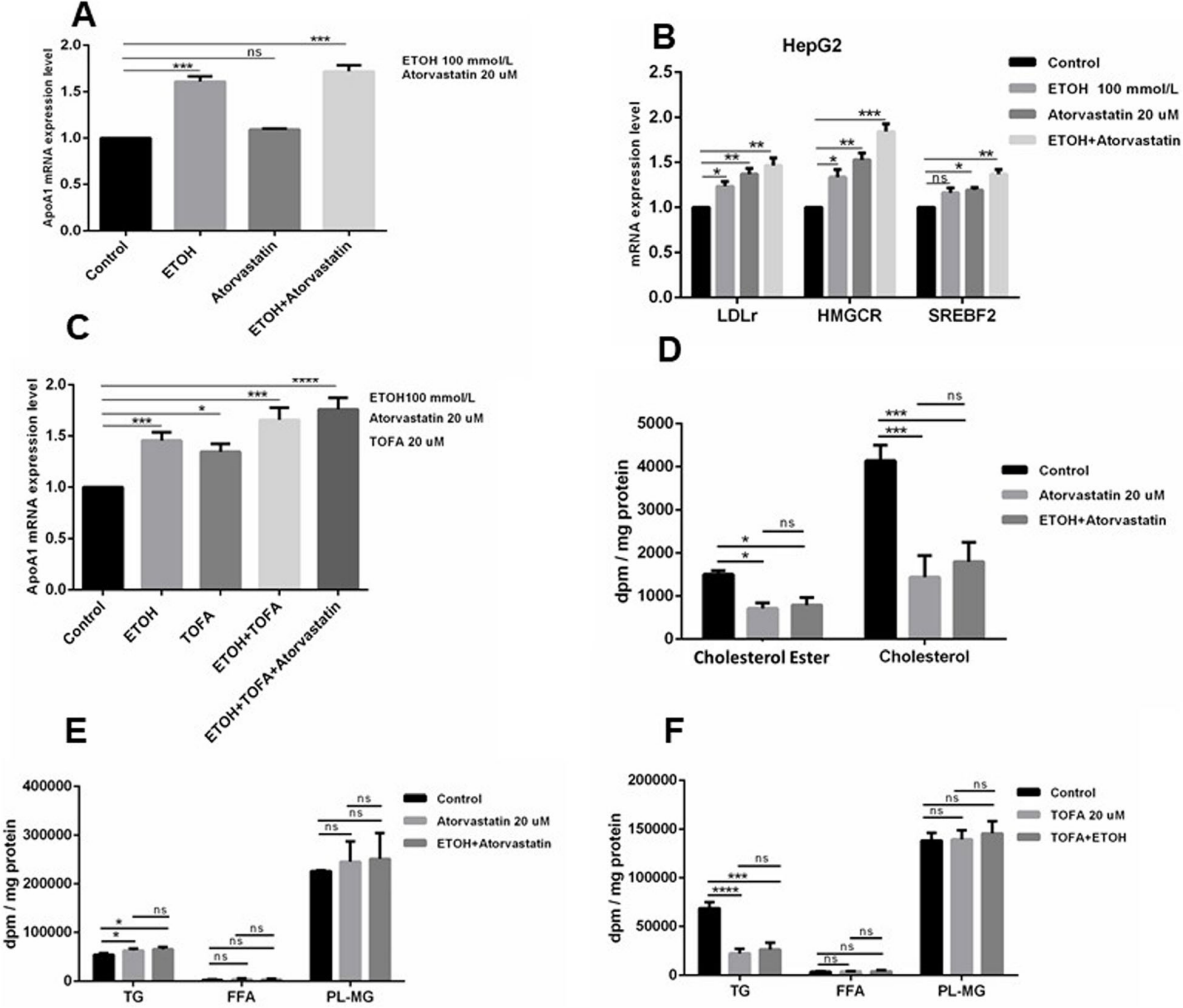
Effects of DNLS inhibitors on induction of *APOA1* RNA expression and on cellular lipid concentration in cells treated with ethanol. Relative RNA expression levels were determined by RT-qPCR as described in Methods. **a** and **b** Expression of *APOA1*, *LDLR*, *HMGCR* and *SREBF2* in HepG2 cells cultured in LPDS medium and treated at the indicated final concentration of ethanol (100 mM) and/or atorvastatin (20uM). **c** HepG2 cells were cultured in standard medium, and exposed to combinations of 100 mM ethanaol, 20 uM atorvastatin, and/or 20 uM TOFA as per Materials and Methods. The error bars represent the SD from the mean of five assays of an individual experiment. *Significant difference, ethanol treatment versus untreated control (1 way ANOVA with multiple comparisons, n = 5; *P < 0.05, **P < 0.01, ***P < 0.001 vs. control). For the effect of DNLS Inhibitors on cellular lipid concentration. HepG2 cells were cultured 21 days in DMEM+ 10% FBS for differentiation, after O/N starvation, cells were incubated with [^14^C] oleic acid and treated with atorvastatin (20 uM) or ETOH (100 m M) + Atorvastatin (20 mM) for 24 h (**d** and **e**). For the **f** cells were treated with TOFA (20 uM) or ETOH (100 m M) + TOFA (20 mM) for 24 h with the concentration indicated of ETOH. After the incubation, lipids were extracted, separated by thin-layer chromatography and quantified as described in Materials and Methods. Data were analyzed as dpm/mg of total protein and represent means±SD for *n* = 3 independent experiments. *P < 0.05, **P < 0.01, ***P < 0.001 vs. control

As shown in Fig. 3a, ethanol continued to upregulate *APOA1* RNA in HepG2 cells in the presence of atorvastatin. The effect was not dependent on the presence of lipoproteins in the culture medium, as the same results were obtained with standard medium containing fetal bovine serum or serum-free LPDS medium (data not shown). The statin alone had no significant effect on *APOA1* expression in the absence of ethanol. The biological activity of the statin was confirmed by assessing de novo cholesterol synthesis directly (Fig. 3d), and by confirming the expected upregulation of RNA from the LDL receptor and HMG Coenzyme A reductase genes (Fig. 3b). These genes are documented to be induced at the RNA level by statins.

TOFA is a potent acetyl coenzyme A carboxylase inhibitor, blocking fatty acid synthesis [37]. As with atorvastatin, addition of TOFA had no inhibitory effect on the upregulation of *APOA1* RNA by ethanol (Fig. 3c). TOFA alone produced a modest, not statistically significant increase in both basal and induced *APOA1* RNA.

To confirm the biological activity of these two inhibitors on cell metabolism, lipid biosynthesis was assayed after pre-labelling cells with ^14^C-oleic acid, which is used as a precursor of several types of lipids. As shown in Fig. 3d, e, f, the ^14^C label from oleic acid was incorporated in cholesteryl esters (CE), free cholesterol (C), triglycerides (TG) and phospholipid-monoglyceride (PL-MG). There was no measured incorporation of label into free fatty acids (FFA). Addition of ethanol to the cell medium led to modest increases in de novo synthesis of cholesterol and cholesteryl esters, triglycerides and phospholipid monoglycerides (data not shown). It should be recalled that the measured lipid synthesis here reflects label originating from the added oleic acid; nothing can be inferred regarding the fate of the ethanolic carbons, although as noted above they are reported to be mostly or completely rapidly converted to acetyl-CoA and ultimately to CO_2_.

As expected, atorvastatin led to a significant decrease in de novo synthesis of cholesterol and cholesteryl esters (Fig. 3d), but not triglycerides or phospholipid-monoglycerides (Fig. 3e). In the presence of atorvastatin, no significant increase in lipid synthesis was observed with addition of ethanol. In contrast to atorvastatin, addition of TOFA led to a significant reduction in de novo synthesis of tryglycerides (as expected), (Fig. 3f), but not cholesterol, cholesteryl esters or phospholipids-monoglycerides (data not shown). Although ethanol addition led to slight increases in synthesis of some of these lipids in presence of TOFA, the increases were not significant.

**Fig. 4:**
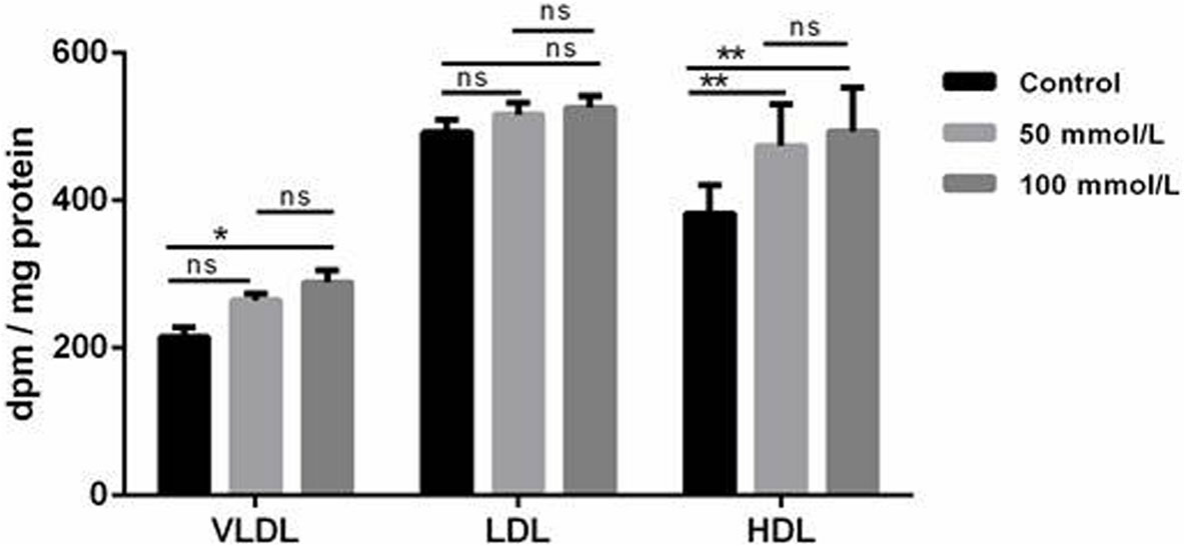
Effect of ETOH on lipoproteins VLDL, LDL and HDL output by HepG2 cells. Following 21 days of differentiation, cells were incubated with [^14^C] oleic acid and ETOH (0, 50, and 100 mM) for 20 h. VLDL, LDL and HDL were isolated by ultracentrifugation according to their specific densities. Radioactivity incorporated into each fractions was further determined. Data were analyzed as dpm/mg of total protein and represent means±SD for n = 3 independent experiments. *P < 0.05, **P < 0.01 vs control

In addition to causing a slight increase in label incorporation into lipids, ethanol also led to a measurable increase of label in HDL particles purified from the treated HepG2 cells (Fig. 4). A smaller increase was also observed for label in VLDL, but no effect on LDL was observed.

### Ethanol upregulates other lipid pathway genes in HepG2 and Caco-2 cells

**Fig. 5:**
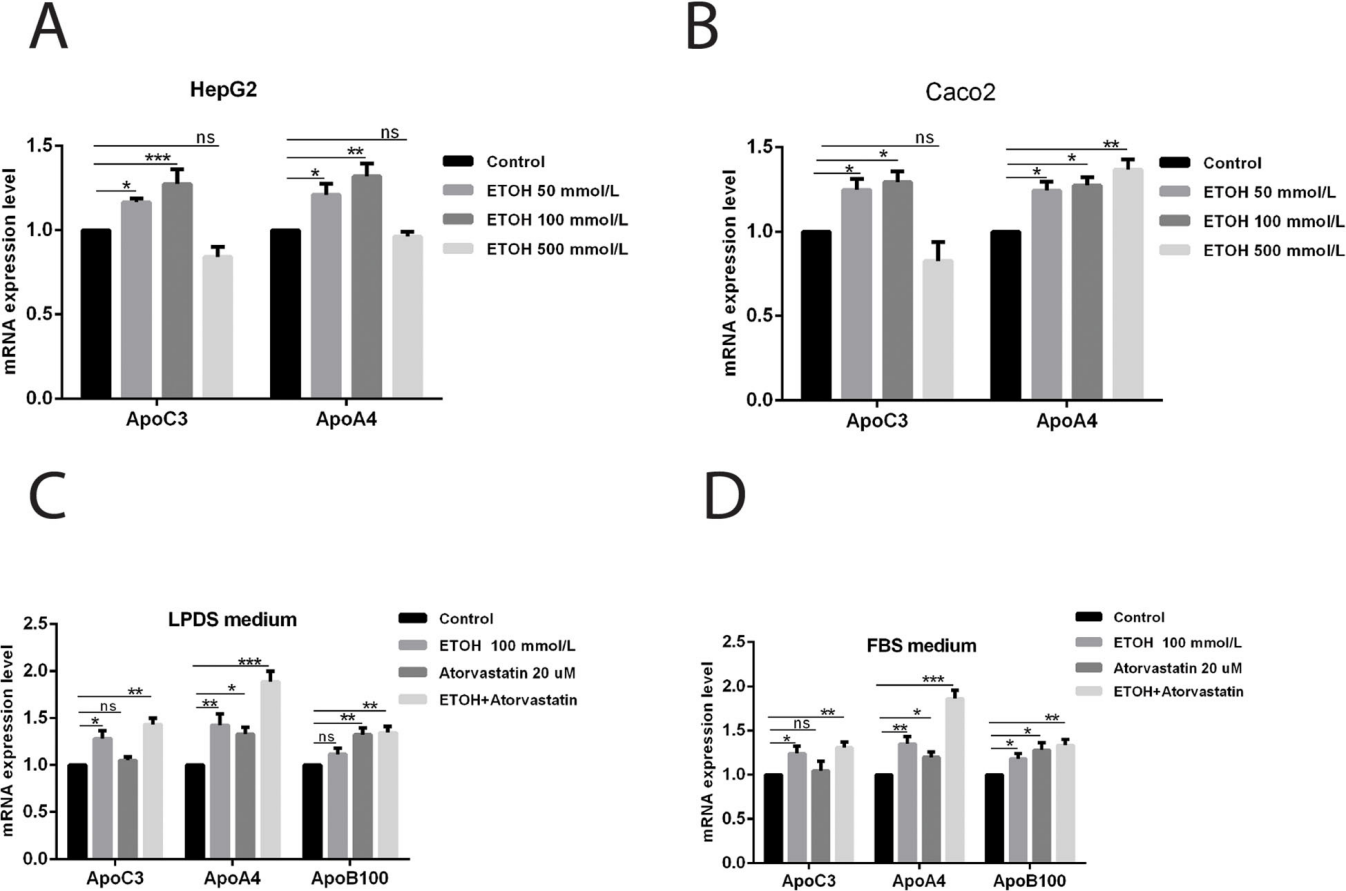
Effects of ethanol on expression of genes involve in the cholesterol biosynthesis pathway. RNA levels of the indicated genes were determined by RT-qPCR as described in Methods. **a**, **b***APOC3* and *APOA4* RNA levels were compared between cell types HepG2 and Caco-2 respectively, treated with the indicated final concentrations of ethanol. **c**, **d***APOC3*, *APOA4* and *APOB* gene expression were compared between standard medium (FBS) and lipid-depleted medium (LPDS), treated with the indicated concentrations of ethanol and/or atorvastatin. The error bars represent the SD from the mean of five assays of an individual experiment. *Significant difference, ethanol treatment versus untreated control (1 way ANOVA with multiple comparisons; n = 5; *P < 0.05, **P < 0.01, ***P < 0.001 vs. control)

A complete transcriptomic analysis of the effects of ethanol in HepG2 cells was beyond the scope of this investigation, however qRT-PCR was used to examine expression of other key lipid pathway genes after exposure of cells to ethanol: *APOC3, APOC4, LDLR, APOB, HMGCR, SREBF2*. Ethanol in the cell medium induced modest but measurable increases in the RNA of all of these genes in HepG2 cells (Figs. 3b and 5; *SREBF2* upregulation did not reach statistical significance with ethanol alone but see below). Unlike *APOA1*, induction of RNA from *APOC3* and *APOA4* was also observed in Caco-2 cells (Fig. 5b). Similarly to *APOA1*, induction of *APOC3*, *APOA4* and *APOB* was observed in cells treated in either FBS or LPDS medium (Fig. 5c, d).

Next the effect of adding atorvastatin to the cell medium on expression of the other genes was examined, alone or together with ethanol. As expected and as noted above, the statin increased expression of the *LDLR* and *HMGCR* genes with or without ethanol, as the cells attempt to compensate for reduced de novo cholesterol biosynthesis caused by inhibition of the HMG-CoA enzyme (Fig. 3b). Induction of *APOB* expression was also observed in response to the statin (Fig. 5c). Expression of *APOC3* was not affected by addition of the statin, however somewhat unexpectedly expression of *APOA4* measurably increased both in FBS or LPDS medium (Fig. 5c, d). Expression of *SREBF2* was upregulated and statistically significant in the presence of ethanol plus statin (data not shown). The effects of ethanol and statin in the medium appeared either independent or potentially additive for these genes.

## Discussion

Our major findings are 1) that currently available HepG2 hepatoblastoma cells in culture show upregulation of *APOA1* gene expression at both the protein and RNA levels, in response to moderate ethanol addition to culture medium; 2) that this response is likely at the level of transcription; 3) that this response is not dependent on de novo cholesterol or fatty acid biosynthesis; 4) that the stimulatory effect on *APOA1* expression is not observed in intestinal-derived CaCO2 cells; and 5) that similar effects on gene expression are observed for a variety of other lipoprotein-related genes of interest. The basic stimulatory effect of ethanol treatment on *APOA1* expression was originally documented in a handful of papers two decades ago, but to our knowledge there have been no subsequent studies of the molecular mechanism underlying the effect. Our cells were freshly obtained from the ATCC, indicating that this very interesting effect is still observed in currently available cells, and thus remains accessible for mechanistic studies, which have been initiated in this report.

The induction of *APOA1* expression by ethanol was both dose- and time-dependent. The basic observation is extremely robust, having been repeated many times with multiple plate replicates in each individual experiment. Although there was some variability in the magnitude of response, typically maximum increase in *APOA1* RNA was 50% (1.5x) above the baseline of untreated cells. While modest, this increase was statistically significant, and is more than twice the typical 10–20% increase in plasma HDL cholesterol in human subjects on a moderate alcohol consumption regime. Moreover, ethanol modestly increased production of HDL and VLDL but not LDL in the HepG2 cells, suggesting that the upregulated ApoA1 protein was biological active to be incorporated in HDL. The effect was not dependent on the presence of lipoproteins in the culture medium, as the same results were obtained with standard medium containing fetal bovine serum or serum-free LPDS medium. The addition of α-amanitin, not previously reported, prevented the induction of APOA1 expression, consistent (though not definitively proving) that regulation of *APOA1* RNA by ethanol is at the transcriptional versus posttranscriptional level. There is no evidence in the literature to suggest that *APOA1* expression is regulated by differential turnover in hepatocytes at the protein or RNA level. Interestingly, intestinal-derived Caco-2 cells did not upregulate *APOA1* in response to ethanol, perhaps because such cells are normally involved in metabolite transfer but not HDL metabolism.

**Fig. 6:**
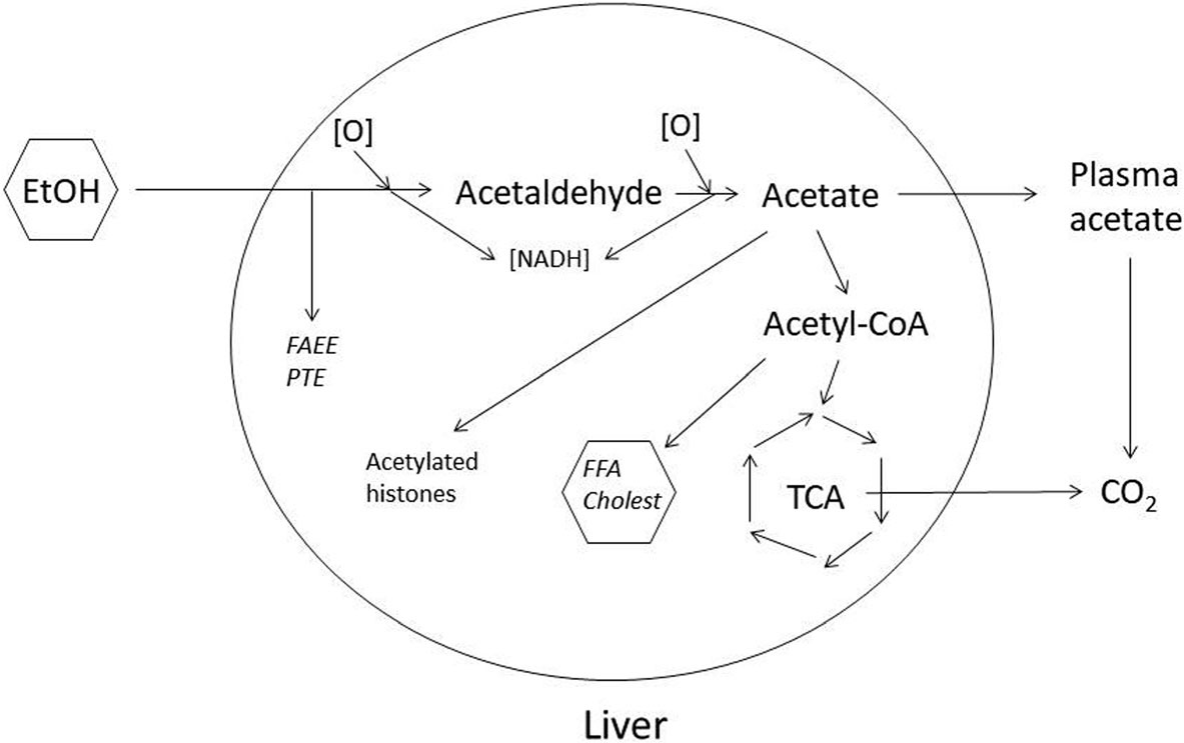
Major metabolic pathways of ethanol in humans. (Adapted from [23, 36, 38]). Orally ingested alcohol (EtOH) is rapidly oxidized in the liver to acetaldehyde, primarily by alcohol dehydrogenase(s), and secondarily by the microsomal ethanol oxidizing system (MEOS, including cytochrome P450 CYP2E1) or by peroxisomal catalase. Acetaldehyde is further rapidly oxidized to acetate, which is either converted directly to acetyl-CoA in the liver, or released to plasma, from where it is ultimately metabolized primarily to CO_2_ in peripheral tissues. Most ethanolic carbon is ultimately released as CO_2_. A small fraction of ethanolic carbon in acetyl-CoA is converted to free fatty acids (FFA) or cholesterol (cholest) via de novo lipid synthesis. A small fraction of ingested ethanol may be non-oxidatively metabolized to fatty acid ethyl ester (FAAE) or phosphatidyl ethanol (PTE). Acetyl-CoA synthetase 2 (ACSS2) also transfers acetate to chromosomal histones [39]. Octagons around EtOH and FFA/cholest denote that inhibitor studies indicate that these are not candidates for primary signaling molecules for ApoA1 upregulation

The metabolism of ethanol in living humans, animal models, and cultured cells, has been extensively studied and is reasonably well understood (Fig. 6). Ethanol is quickly oxidized to acetaldehyde in the liver. Generally the main pathway of this reaction is via the family of alcohol dehydrogenases; however these genes are not highly expressed in HepG2 cells, which use a secondary pathway for ethanol metabolism, the microsomal ethanol oxidation system or MEOS. A previous report documented that inhibition of the MEOS by metyrapone blocked the upregulation of ApoA1 protein by ethanol in HepG2 cells, indicating that ethanol is not itself the primary signaling molecule in the response. Importantly, the lack of alcohol dehydrogenase activity in HepG2 cells (unlike in normal hepatocytes) is unlikely to impact our findings or interpretation, since conversion of ethanol to acetaldehyde is as efficient in HepG2 cells as in intact liver, and the effects on *APOA1* expression appear to involve steps downstream of acetaldehyde synthesis. Acetaldehyde is a toxic molecule, and is quickly metabolized to form acetate via the acetaldehyde dehydrogenases. Acetate can undergo several further reactions, the major one being formation of acetyl-CoA, either in the liver or in peripheral tissues via transfer of acetate in plasma. Thus, together with previous work, our results suggest that the primary signaling molecule in this system may be either acetaldehyde, acetate or acetyl-CoA. Given the rapid turnover of acetaldehyde, and the very general metabolic properties of acetyl-CoA, one may speculate that acetate is the direct signaling molecule leading to upregulation of APOA1. In one experiment this was tested directly, however at the maximum non-toxic concentration of acetate in the culture medium, there was no effect on ApoA1 protein in our HepG2 cells; the reason for this is unclear; possibly the maximum non-toxic concentration of acetate that could be added to the medium was insufficient to induce the effect, or conceivably acetate generated endogenously from ethanol may have different actions than acetate imported from the extracellular environment. Interestingly, one of the enzymes that converts acetate to acetyl-CoA, ACSS2, also transfers acetate to histone lysines in chromatin [39]. It was also recently shown in mice that ethanol-derived carbon is found in acetylated histones in the brain [40]. This suggests the intriguing possibility that ethanol may regulate gene expression through chromatin remodeling. In addition to these pathways, a small fraction of ethanol may be metabolized non-oxidatively to either fatty acid ethyl ester or phosphatidyl ethanol [38]. It seems unlikely that either of these would play a role in regulating ApoA1 gene expression, but this may be explored in further research. Finally, it is worth noting that the oxidation of ethanol leads to a significant, though transient, increase in cellular NADH pools in liver cells. A large change in oxido-reductive potential could also lead to changes in gene expression, although it is not obvious why *APOA1* would be upregulated by increased cellular reducing potential.

A potential concern in our study is the difficulty in relating concentrations of ethanol in our cultured cells, versus in the hepatocytes of human subjects ingesting alcohol orally over very different exposure times (hours versus weeks). Notwithstanding this issue, the similar levels of the relevant biological response of our cultured cells compared to human subjects, in terms of *APOA1* upregulation (50% over 24 h in cultured cells) versus total HDL-C upregulation (15% over several weeks in humans), is sufficient to make this an attractive model system. Once specific primary targeting molecules and transcriptional regulatory elements of the *APOA1* and the other lipoprotein genes have been identified in vitro, these can be explored in more complex systems such as primary hepatocytes or humanized mice. It should be kept in mind that mice normally have very high HDL to LDL ratios and are a poor default model for HDL lipid studies unless genetically modified.

Recent studies of genetic population variation influencing HDL-C, as well as the results of clinical trials of new potential therapeutic entities (particularly inhibitors of cholesteryl ester transfer protein, CETP), have recently called into question the cardioprotective role of HDL-C, or at least have suggested that the relationship to CVD risk reduction is more complex than simply the absolute concentration of HDL-C in the blood [41, 42]. Probably the metabolic flux of HDL as well as the detailed molecular composition of various subtypes of HDL-like lipoprotein particles, are more directly relevant biomarkers [43, 44]. Nonetheless, there is continued interest in HDL metabolism as a potential therapeutic target in CVD. Therefore, a better understanding of the molecular basis of *APOA1* upregulation by ethanol is of potential value to the pharmaceutical industry.

## Conclusions

This report describes the first mechanistic study of regulation of apolipoprotein A1 gene expression in hepatocyte-like cells exposed to moderate alcohol concentrations. Epidemiologic studies have documented the beneficial effects of moderate alcohol consumption on heart disease in general, and serum HDL-cholesterol in particular, however the mechanisms underlying these effects are unknown and have not been extensively researched. HepG2 hepatoblastoma cells were used as a model for liver function, as these cells have previously been shown to upregulate *APOA1* expression when exposed to moderate ethanol concentration in the culture cell medium; moreover, HepG2 cells are widely accepted as a model for liver cells in culture. It is shown here for the first time that this regulation is most likely at the level of transcription, as treatment of cells with the RNA polymerase II inhibitor α-amanitin eliminated the upregulation. In contrast, treatment with the de novo lipid biosynthesis inhibitors atorvastatin or TOFA had no effect on *APOA1* upregulation by ethanol, demonstrating that the effect is not due to new cholesterol or fatty acid synthesis. Further, *APOA1* was not upregulated by treatment of intestinal-derived Caco-2 cells, indicating that the effect is at least partially cell- or tissue-specific. As previous studies have excluded ethanol itself as the primary signalling molecule, it is suggested that either acetate or acetyl-CoA may be the primary signalling molecular upregulating *APOA1* expression.

## Data Availability

All experimental results are available for interested readers. There are no specialized materials employed in the study.

## Abbreviations

*APOA1*: Apolipoprotein A1
*APOA4*: Apolipoprotein A4
*APOC3*: Apolipoprotein C3
*APOB*: Apolipoprotein B
*SREBF2*: Sterol regulatory element-binding protein 2
*HMGCR*: Hydroxymethylglutaryl-CoA reductase
*LDLR*: Low Density Lipoprotein Receptor
*CETP*: Cholesteryl Ester Transfer Protein
LDL or LDL-C: Low-Density Lipoprotein Cholesterol
HDL or HDL-C: High-Density Lipoprotein Cholesterol
VLDL: Very-Low Density Lipoprotein
DNLS: de novo lipid synthesis
